# Physical Considerations for In Vitro ESWT Research Design

**DOI:** 10.3390/ijms23010313

**Published:** 2021-12-28

**Authors:** Cyrill Slezak, Roland Rose, Julia M. Jilge, Robert Nuster, David Hercher, Paul Slezak

**Affiliations:** 1 Department of Physics, Utah Valley University, Orem, UT 84059, USA; CSlezak@uvu.edu; 2 Ludwig Boltzmann Institute for Experimental and Clinical Traumatology, AUVA Research Center, 1200 Vienna, Austria; roland.rose@protonmail.com (R.R.); julia.jilge@trauma.lbg.ac.at (J.M.J.); david.hercher@trauma.lbg.ac.at (D.H.); 3 Austrian Cluster for Tissue Regeneration, 1200 Vienna, Austria; 4 Department of Life Science Engineering, University of Applied Sciences Technikum Wien, 1200 Vienna, Austria; 5 University of Veterinary Medicine Vienna, 1210 Vienna, Austria; 6 Department of Physics, University of Graz, 8010 Graz, Austria; ro.nuster@uni-graz.at

**Keywords:** shockwave therapy, regenerative medicine, sedimentation, in vitro

## Abstract

In vitro investigations, which comprise the bulk of research efforts geared at identifying an underlying biomechanical mechanism for extracorporeal shock wave therapy (ESWT), are commonly hampered by inadequate descriptions of the underlying therapeutic acoustical pressure waves. We demonstrate the necessity of in-situ sound pressure measurements inside the treated samples considering the significant differences associated with available applicator technologies and cell containment. A statistical analysis of pulse-to-pulse variability in an electrohydraulic applicator yields a recommendation for a minimal pulse number of *n* = 300 for cell pallets and suspensions to achieve reproducible treatments. Non-linear absorption behavior of sample holders and boundary effects are shown for transient peak pressures and applied energies and may serve as a guide when in-situ measurements are not available or can be used as a controllable experimental design factor. For the use in microbiological investigations of ESWT we provide actionable identification of common problems in describing physical shockwave parameters and improving experimental setups by; (1) promoting in-situ sound field measurements, (2) statistical evaluation of applicator variability, and (3) extrapolation of treatment parameters based on focal and treatment volumes.

## 1. Introduction

Over the last 30 years, extracorporeal shock wave therapy (ESWT) has established itself as a viable treatment option for a wide range of clinical indications. It has distinguished itself for its safe, non-invasive approach in regenerative medicine [1] when it adheres to established protocols. A well-established wide range of indications like non-union fractures, some tendinopathies, and chronic non-healing wounds is continuously expanded by new experimental investigations into novel indications in urology, peripheral nerve regeneration, spinal cord injury, and trans-cranial applications [2,3,4,5,6,7,8,9,10,11].

Clinical successes lead to the massive need for bedside-to-bench research seeking insights into the underlying biomechanical interactions, which constitute the foundation of the respective treatments’ regenerative potential. Active in vitro experimentation alongside pre-clinical animal models are establishing a direct link between physical parameters of shockwaves and corresponding biological responses, which can be further correlated with clinical outcomes. This search for a fundamental mechanistic understanding of underlying regenerative mechanisms, however, remains elusive [12,13,14]. Possible working mechanisms have been proposed and investigated [15,16], but many basic science results have been shown to suffer from poor-documentation and irreproducibility [17,18].

A complete and detailed description of the applied acoustical fields is essential for the investigation of mechanobiology and cellular mechanosensing. Currently, there is no clear causal link between specific physical parameters such as peak pressure or pulse energy and any specific cellular response, but rather only a cumulative effect of the entire shockwave. The characterization of the applied sound fields is often incomplete, and the used parameters are restricted to the technical settings of the applicators [17,19]. This is particularly problematic as the requirements for approval of medical shockwave devices by regulatory agencies are based on dated standards derived from ultrasound devices and applications [20]. There, one of the main safety aspects for patients is the time-averaged energy deposition within the tissue. However, the clinical use of ESWT is limited to a few pulses (usually less than 10 per second) rather than a continual signal, and as such, the total power is insignificant at comparable pulse pressures. Therefore, documented settings provide researchers with upper safety thresholds but only limited information about actually applied therapies.

Most commonly reported parameters describing in vitro setups have focussed on peak pulse pressure, pulse intensity integral (also referred to as energy flux density), and pulse repetition frequency [21]. These can be thought of as envelope parameters, providing ranges and summative reference values of the pressure-time waveform but do not allow for any insight into its actual shape. Additional corresponding geometric sound field parameters which may be readily obtained indirectly through the listed manufacturers include the −6 dB and the 5 MPa zones. These correspond to focal volumes of peak pressures exceeding 50% of the maximum peak pressure and those exceeding the 5 MPa threshold, respectively [22]. It is noteworthy that all these geometric descriptors only provide summative (for energies) or maximal (for pressures) values and none of the waveforms (time evolution of the pressure) themselves. As such, all observed biological in vitro effects have only been correlated to this limited set of physical parameters, absent any indication of causality or even suggestion of a physical mechanism [17,23].

This paper provides a systematic approach to in vitro research design using electro-hydraulic ESWT in adapting the physical characterizations of lithotripter experiments into the low- to mid-energy regime. In particular, we present a complete description beginning from a visualization of the applied pressure waves, provide a statistical analysis of the applied treatments, evaluate the impact of the cell containers on the pressure field, and validate the use of cell pellets and sedimentation in suspension samples. The detailed approach allows for the adoption by research groups with limited technical (physical) experience and resources while maintaining a controlled treatment environment. This will hopefully lead to an increase in the reproducibility of future biological experiments using ESWT.

## 2. Results

Regulatory compliant free-field measurements (such as IEC 61846) for electrohydraulic devices can differ widely from the actual use of the device in in vitro setups. In full therapy sessions, up to several hundred pulses are applied to the study sample in rapid succession (application frequency). Of particular interest in this setting is the pulse-to-pulse variability within the therapy. Due to both the regulatory requirement and their predominant reporting in the literature, the pulse peak pressure $p_{max}$ and peak integral intensity PII, often referred to as energy flux density over the focal zone, are of special interest. Although *PII* can be differentiated only taking into account positive or negative wave-components, we consider the total waveform and define

$$PII=\frac{1}{\rho c}\int^{\;}p^{2}\left(t\right)dt$$

where ρ is the mass density of the medium, c the speed of wave propagation, and p(t) is the measured time-dependent pressure.

**Figure 1:**
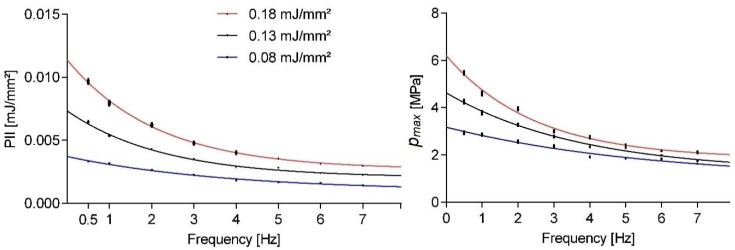
Evaluation of pulse integral intensity (PII) and maximum pulse pressure (*p_max_*) at nominal machine settings at various application pulse repetition frequencies. Error bars shown indicate the standard error of the mean.

Figure 1 shows the pulse repetition frequency dependent peak pressures and PII for a given nominal machine setting. All results shown in this paper indicate the mean statistical values and associated standard error of the mean. Therapy sessions comprised 300 shots utilizing an OP155 applicator as evaluated by a point measurement inside the focus volume at a fixed location for all measurements. The three evaluated energy settings reflect most of the applicator’s energy range. None of the evaluated parameter means approached the nominative values even in the extrapolated single shot limit (i.e., frequency approached zero). As the application frequency was increased, we observed a strong exponentially decaying sound field strength.

**Figure 2:**
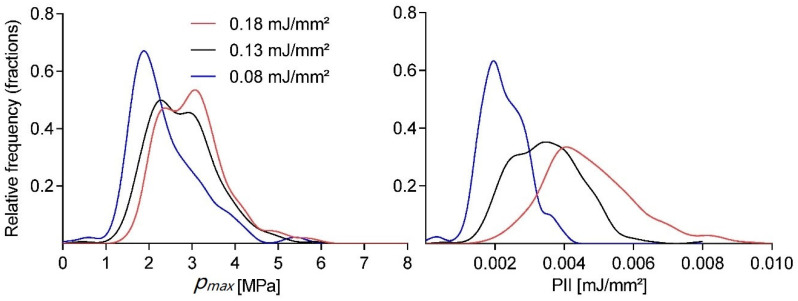
Normalized smooth histogram of 1200 shots for OP155 maximum pulse pressure (*p_max_*) and pulse integral intensity (PII) measurements at three nominal machine settings and 3 Hz application frequency.

Taking a closer look at the variability within single setting runs, Figure 2 shows the normalized smooth histogram for the distribution of pulse peak pressures and energies over 1200 shots at a fixed application frequency of 3 Hz. There was a fairly strong cut-off for peak pressures below 1 MPa regardless of energy setting, while comparable high peak pressures could also be found in the high-end tail. There was a significant overlap between the three energy settings, but a distinct separation of the means, as already documented in Figure 1. 

The large variation of the observed individual shockwave pulses raises the question as to whether the two subsequent treatments are statistically comparable. In order to estimate the relative error corresponding to a confidence interval (CI) of any expected mean value for a physical parameter from our stochastic generator for a given sample size of applied shocks, we employed a Monte Carlo (MC) approach implemented in Mathematica (Version 12.0, Wolfram, Champaign, IL, USA). 

We determined the absolute error $\varepsilon \geq \left|\;\bar{x_{n}}-\;µ\right|$ for a given CI by applying the central limit theorem

$$\mu =\underset{n\to \infty }{\lim}\left(\bar{x_{n}}=\frac{1}{n}\overset{n}{\underset{i=1}{\sum }}x_{i}\right)$$

based on n random therapy samples $Y_{n}$ which are drawn from an experimentally determined distribution Y with mean μ and standard deviation $\sigma^{2}$. Since $\mu_{\bar{Y}}=\mu$, we estimate the error ε for the 100 × (1 − α)% confidence intervals by the corresponding standard deviation of the absolute errors of $\bar{Y}\left(Y_{1,\;}Y_{2,\;}Y_{3,\;}\cdots Y_{n\;}\right)$. Minimal therapy sample sizes could now be obtained by matching the desired error with the appropriate interpolated CI curve.

**Figure 3:**
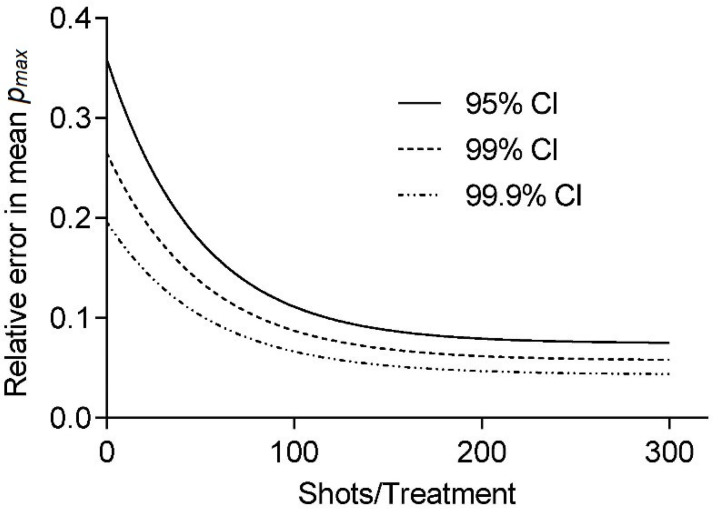
The estimated relative error in the mean maximum pulse pressure (*p_max_*) of a treatment session. Computed results for various treatment shot sizes of a Mont Carlo simulation based on an experimental probability distribution for three different confidence levels are shown.

Figure 3 shows the relative error $\varepsilon_{rel}$ estimates in the peak pressure for CIs of α=0.05, 0.01, 0.001 as a fraction of the sample mean μ. We obtained a probability density function (pdf) from the normalized smooth histogram interpolation of 1200 pressure measurements for an OP155 un-focused applicator at 3 Hz and an energy setting of 0.08 mJ/mm^2^. The MC calculation was based on n=100,000 steps for each therapy size. The results indicate a significantly larger minimal sample size $n_{ss}$ for the desired error ε as would be obtained by assuming a normalized distribution of pressures

nss≥(z1−α2 σε)2

The results presented up to this point have illustrated the inherent variability of the underlying free-field pulse propagation utilizing a water bath set up in the absence of any samples in the system. In studying the correlation between biological effects and physical parameters, one aim is to provide the aforementioned characterization of the sound field while simultaneously mimicking pre-/clinical indications. As such, it is desirable to avoid variations in medium impedance for the study of soft-tissue applications and a controlled approach to introduce reflective interfaces such as in the proximity of air and/or bones. One very common approach is the use of established cell and tissue sample containers in the form of Falcon tubes or flasks. The utilized plastic and any additional cell medium (i.e., in the study of cell suspensions) are similar in impedance to water. Such an approach will largely maintain the free field geometry of the reference bath measurements discussed so far.

**Figure 4:**
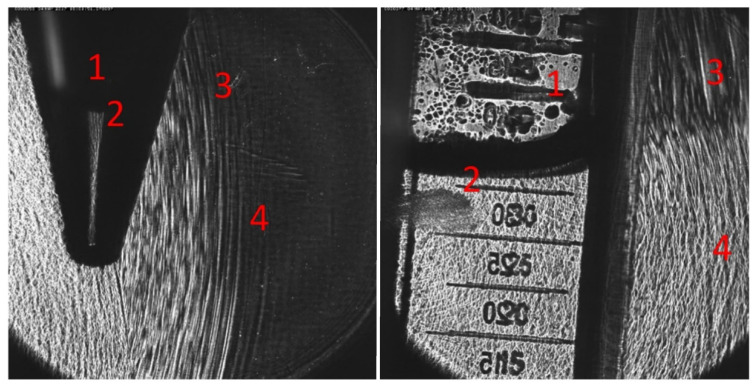
OPCD images of an electrohydraulic generated shockwave front incident on a sample filled 10 mL Falcon tubes (**left**) and a 25 cm^2^ cell culture flask (**right**). No wave propagation is observed through air compartments (1) and reflected (2), diffracted (3), and transmitted (4) wave-components are clearly visible about the sample-air interface in the container.

However, in the presence of an air interface, the vicinity of the sample and/or the focal zone of the applicator results in pulse reflection and refraction at the said interface. In Figure 4, OPCD images of the pulse wave-front are shown visualizing the altered sound fields of partially filled containers exhibiting many of the key features: (1) There was no propagation of the pressure pulse through the air column above the sample, (2) essentially perfect reflection of the oncoming wave at the air-interface, (3) diffraction effects expanding into the volume behind the air volume, and (4) almost full propagation of the incident wave through the sample. Thus, the resultant wave pulse treating the sample was fundamentally altered by the introduction of the air volume within the container.

**Figure 5:**
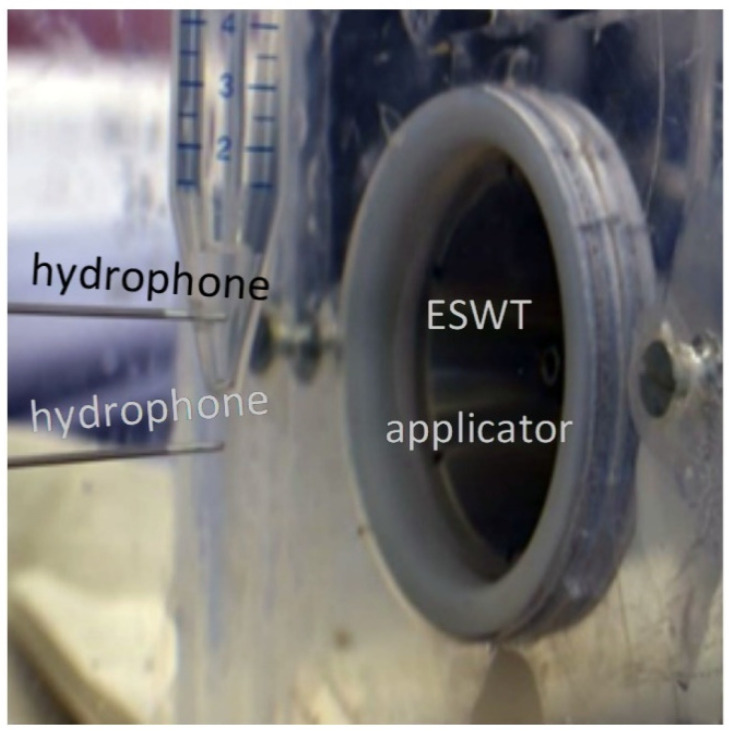
Representative setup of evaluating wave-pressures in the focal zone of the shockwave applicator either inside the sample (**top** hydrophone) or as a free-field reference measurement (**bottom** hydrophone). Depicted off-axis positions are for illustration purposes only.

In order to quantify the physical sound parameters of the altered shockwave, we have to be able to probe the sound field within the sample, thus accounting for effects of the container, sample, and air volume proximity. For this purpose, the hydrophone was placed in the center of the sample volume such that it coincided with the free-field focal point of the applicator. Figure 5 depicts the inserted pressure probe through a small drill hole in the back of the container at the center height of the sample. 

**Figure 6:**
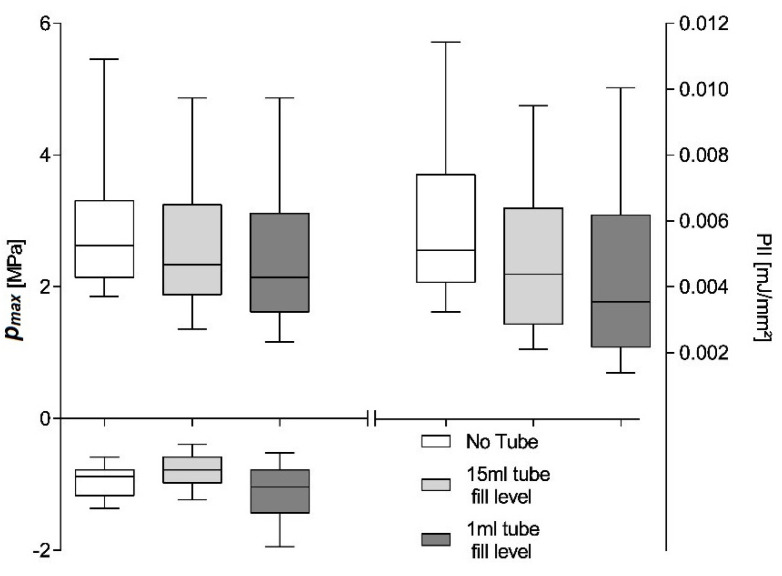
Comparison of positive and negative peak pressure and pulse integral intensity within a fully and partially filled (1 mL) 15 mL Falcon tube to a free-field reference (no tube). All sample medians are highly statistically different (Kruskal–Wallis, *p* << 0.001).

The in situ placement of the hydrophone in the in vitro setup allows for the systematic investigation of the effects of the sample container and its fill-level on the shockwave. Figure 6 shows maximum pulse pressure (*p_max_*), maximum tensile pressure (*p_neg_*), and pulse integral intensity (PII) for a 600 shot treatment using the OP 155 un-focused applicator at a nominal energy setting of 0.18 mJ/mm^2^ and an application frequency of 3 Hz. The hydrophone placement is identical for the three investigated scenarios within the measured applicator focal volume close to the geometric focal point. We compared the shockwave parameters of the free-field measurements to those inside a partially (1 mL) and fully filled 15 mL Falcon tube. The hydrophone was fixed relative to the applicator and placed at the center of the tube at a height of the 0.5 mL fill-level.

The free-field reference measurements yield a median maximum pulse pressure *p_max_* = 2.975 ± 0.04909 MPa, maximum tensile pressure *p_neg_* = −0.93 ± 0.011 MPa, and pulse integral intensity PII = 0.006 ± 0.0001 mJ/mm^2^. Introducing the cell container reduces all parameters to *p_max_* = 2.499 ± 0.0492 MPa, *p_neg_* = −1.119 ± 0.0184 MPa, and PII = 0.004 ± 0.0001 mJ/mm^2^ as a direct result of the tube walls and is independent of the fill level. All sample medians are highly statistically different (Kruskal–Wallis, *p* << 0.001) and individually distinguishable (subsequent post-hoc Dunn’s multiple comparisons). Non-parametric analysis was chosen due to the nature of the distribution of generated pulses (see Figure 4) and a subsequent comparison of the median as the distributions were similar. Note, the latter may not be applicable if different applicator energy levels are compared, in which case results should only be interpreted in terms of dominance. A comparison of means would furthermore be susceptible to high energetic outliers, and the possible existence of a threshold pressure for biological effects (i.e., 5 MPa zone) furthermore suggest a rank comparison.

The shift of the parameters median to lower values can be attributed to a predominant loss of higher-pressure pulses, as evident by the lower median within the interquartile range. By reducing the contained water volume in the Falcon tube to 1 mL and thus bringing the contained air interface within the treatment zone, the tensile pressure is essentially doubled due to the phase inversion incurred at the open boundary reflection. It is important to note that this is due to the hydrophone placement with the interface’s pressure zone and is absent in the filled tube where the air interface remains outside the treatment zone.

**Figure 7:**
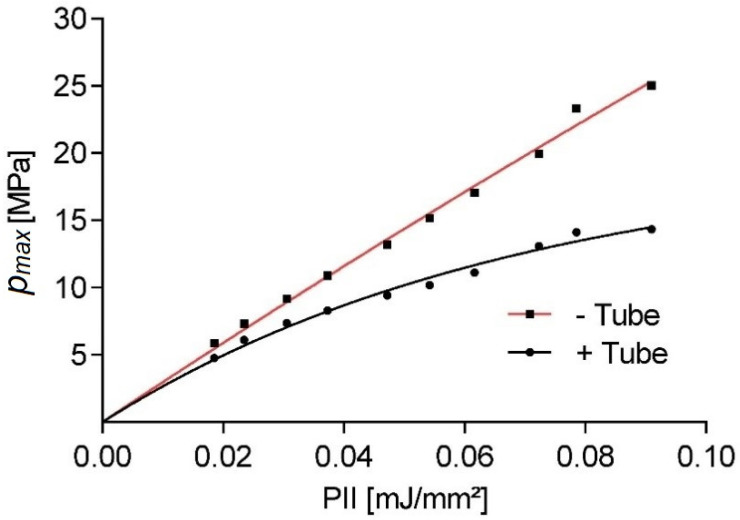
Comparison of measured peak pulse pressure inside a water-filled Falcon tube (black) and a free-field measurement (red) vs. the nominal energy setting of an electromagnet applicator.

To gain a better understanding of the pressure-dependent pulse attenuation, the peak pressure inside a Falcon tube was investigated. Similar work has been previously done using lithotripter [24], but significantly larger focal lengths and energies make the results only marginally translatable. Here we use an electromagnetic applicator as the reproducible pulse form generated by this type of applicator allows for a systematic investigation of the energy-dependent boundary/transmission effects. A similar setup to the previous experiment only utilizing completely filled 15 mL Falcon tubes is used to eliminate any water/air boundary effects. Figure 7 shows the applicator energy-dependent reduction of peak pressures within a Falcon tube in comparison to the free-field value. The hydrophone placement is held constant throughout the experiment, thus not accounting for any possible lensing/refocusing effects of the inserted tube. The measurements show a significant reduction of peak pulse pressure of 74.8% for higher pulse pressures, while only small changes are observed at lower pressures. The observed non-linear behavior is in line with the expected power law-dependent absorption of the tube-wall material (15 mL polypropylene centrifuge tubes, Greiner Bio-One GmbH, Kremsmünster, Austria).

The results up to this point have clearly illustrated the necessity for in situ evaluation of applied sound fields for in vitro experiments. However, this point wise evaluation of the acoustic pressure fields may only be correlated to specimens in the same vicinity which further need to remain there for the duration of the treatment.

One frequently used approach to spatially isolate a sample is to use gravitational cell sedimentation within a medium for localizing the target cells at the tip of a Falcon tube for best ESWT placement. The prevailing assumption has been the resulting localization remains intact despite the mechanical impulses from the shockwave even though significant motion has been observed in larger pellets [17]. To investigate the viability of this approach, time-dependent cell-density measurements of an initially well-mixed sample were taken at 10 min intervals up to 60 min. Vertical stratification was evaluated from four surface level 50 µL samples in each Falcon tube starting from the top (a) and spaced by subsequently discarding of the top 1 mL of the sample (b–d). The final fifth sample (e) was taken from the bottom of the Falcon tube before discarding the rest. 

**Figure 8:**
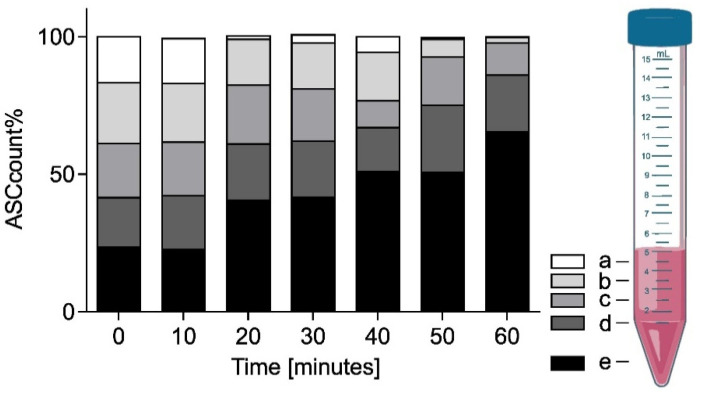
Fractional ASC count over 60 min showing increasing sedimentation within a 5 mL sample. Locations (a–e) correspond to 1-mL increment fill levels.

Figure 8 shows the mean fractional ASC count per fill-height obtained based on a double cell count analysis via Neubauer counting chamber at each time point (*n* = 3 with double readouts). At *t* = 0 min, the cells were in a homogeneous emulsion with an associated probability of finding 20% of the cells at each sample location (a–e). While over the initial 10 min relatively little sedimentation was observed in the bottom layer 23 ± 1.3%, the average cell count doubled (41 ± 11.9%) after 20–30 min. Subsequently, the sedimentation and accumulation of cells in the bottom slows down. After 40–50 min half (51 ± 5.0%) and after one hour two thirds (65 ± 7.8%) of the cells were found in the bottom fraction.

While sedimentation effectively accumulates the bulk of the suspended cells within the bottom 1 mL of the Falcon tube, it is only a viable option for in vitro investigation if no significant mixing occurs due to the shockwave application itself. Two sample groups of *n* = 8 were prepared identically for the sedimentation experiment and rested for a minimum of 40 min, which should render half of the cells in the bottom fraction. Subsequently, the Falcon tube was fixed in a water bath, placing the focal point of the applicator at the center of the bottom 0.5 mL volume. Each group was treated with 300 shockwave pulses at 3 Hz at the applicator’s highest available energy setting to achieve the largest possible agitation. The electromagnetic “Sepia” DUOLITH SD1 (Storz Medical) at an energy level of 0.55 mJ/mm^2^ was used for group 1. The electrohydraulic OE 50 applicator (Dermagold 100; MTS Medical; Germany) at an energy level of 0.27 mJ/mm^2^ for group 2. Stratified samples were taken immediately after the conclusion of the treatment analogously to the methodology described previously.

**Figure 9:**
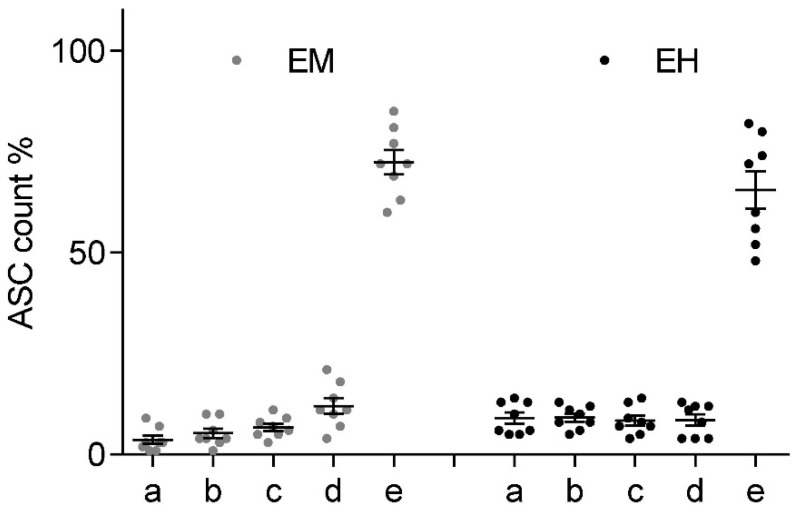
Fractional ASC count by 1 mL incremented fill-height (a–e) within a 15 mL Falcon tube after ESWT. No significant mixing is observed for either electromagnetic (**left**) or electrohydraulic (**right**) shockwave applicators at respective maximum energy settings.

Figure 9 shows the fractional ASC count for each ESWT applicator at each of the 5 height levels within the Falcon tube (a–e). Post-treatment mixing of the sample was observed to be less than in the previously established 40-min sedimentation levels. It should be pointed out that the significantly higher peak pressure of the electromagnetic device was restricted to significantly smaller volume when compared to the electrohydraulic device, while the latter had a sizably smaller peak pressure and a stochastically varying (shifting focal volume) field. However, in both applications, we observed no net redistribution of cells within the suspension.

## 3. Discussion

One of the key challenges in unlocking a mechanistic understanding of ESWT remains a refined conception of the effects physical shockwaves have on biological systems. Experimental in vitro setups strive to provide a homogeneous acoustic pressure field within the treatment zone to facilitate the correlation of observed cellular responses. However, we have once again shown that using electrohydraulic shockwave devices requires an inherent statistical treatment of the acoustical field parameters due to their stochastic generating mechanism. While electromagnetic and piezo applicators provide such a desired highly reproducible field, they lack the possible benefits associated with random variations [25].

Our measurements systematically documented a significant pulse-frequency dependent loss in generated peak pressures and associated energies for electrohydraulic applicators. There is a well-understood effect of generated cavitation leading to peak pressure degradation due to energy absorption and wave reflections. This has been mostly studied for the refocusing high-energy shockwaves (lithotripters) in the treatment zone—the region of concern for in vitro and clinical application [26,27]. However, we expect the highest levels of cavitation bubbles in close proximity of the spark gap and to remain enclosed within the generating applicator. Bubbles produced by the generated shockwave only partially dissolve in the limited time before subsequent shock pulses arrive, thus providing additional nucleation sites for further cavitation. Based on our pulse-frequency-dependent output measurements, there appears to be a cumulative saturation level based on the recovery (dissolve) time, providing a predictive dampening of pressures and energies.

While cavitation effects appear to be systematically dependent on pulse repletion rate, it does not explain the high inter-pulse variability associated with electrohydraulic shockwave sources. This is evident in the statistical spread of pressure and energy measurements using single device settings. The observed outcome is a combination of effects associated with the spark generation, which results in the plasma bubble-induced shockwave formation. For one, any spark discharge between two cathodes is a stochastic process and the subsequent location and shape of the plasma channel and subsequent bubble geometry are indeterminate, arguably exacerbated by the small geometries of handheld applicators. Any deviation from a spherical shockwave centered about the geometric focus of the reflector will result in a blurred refocused image which will have a peak pressure center shifted away from the geometric focus location. This explains the variation in hydrophone peak pressure measurements at the geometric focal point as only occasionally a generated shockwave will refocus at the location of the hydrophone, thus recording maximum peak pressures. For most pulses, the acoustical focus will be found displaced about the geometric one, and the hydrophone picks up only a strongly distant dependent attenuated signal.

For larger studies, it is essential to account for the variability in the generated shockwaves and to assure that within any two or more experimental treatment sessions, the applied pulses are statistically similar. Figure 3 clearly indicates that even at the desired confidence interval CI = 95% and a relative error in the mean peak pressure of $\varepsilon_{rel}=5\%$ requires a minimum sample size $n_{min}=$ 187 pulses to obtain comparable treatments. This is significantly more than would be expected for a normally distributed source with $n_{min}=$ 61. It is unreasonable to obtain a large statistical sample for each machine setting to obtain a reliable probability distribution function as the applicator would show significant degradation of the spark gap electrodes over the course of the measurements. An alternative approach is to use a bootstrapping approach by which to determine the underlying statistics. Obtaining distribution parameters using a pseudo-random re-sampling of the measurement data obtained for a single treatment can provide additional insight. Using this approach to estimate minimum sample sizes on larger data sets (those used in Figure 1), we found large variations in distributions. The comparably small sample size of only 300 pulses, in general, resulted in estimates larger $\varepsilon_{rel}$ given the same CIs. While individual setups can be characterized in this fashion, we recommend, based on our findings, that using no less than 300 pulses is preferential. We argue that while some applicators and/or settings may require more shots, this choice will allow for a high level of confidence for most setups while maintaining a manageable treatment time for short duration protocols.

Once the treatment parameters are decided, nominal free-field pressure and energy reference data for applicator outputs are inadequate for describing in vitro setups. Spatial and temporal characteristics of the shockwave field are affected by individual setups and therefore need to be evaluated in situ. The localized impact of containers, media, and air interfaces can only be estimated through involved numerical simulations but can readily be measured by in vitro research groups. In addition, a direct measurement of the waveform provides additional information lost in the envelope parameters (PII, *p*_max_, *p*_neg_). Pressure gradients, rise and relaxation times, and pulse widths are known to be device-specific and may have an effect on treatment outcomes. PVDF hydrophones allow for a robust, easily implantable, and cost-effective solution, which allows for localized multi-pulse evaluation, which cannot be done by often prohibitively priced fiber optic sensors. While PVDF hydrophones underestimate the negative pressure of the wave at high peak pressures, these energies are unlikely to be evaluated in vitro outside of lithotripsy investigations. 

However, while electromagnetic and piezo applicators provide such a desired highly reproducible field, it exhibits a strong special dependence restricting the recommended treatment zone of the sound field to less than half of the −6 dB focal size in the free field. The resulting, sometimes restrictedly small volume along with the alteration of the local pressure fields due to the incorporation of cell containers requires further scrutinization of any setup and brings into question the sometimes-argued preferentiality of these types of technologies for the use of in vitro investigations [17].

Direct in situ measurements are a great step towards standardization, but additional spatial considerations need to be included as well. Specified parameters for the approximately ellipsoidal focal size are (1) the 5 MPa zone wherein peak sound field pressures exceed this nominal threshold pressure and (2) the −6 dB zone, where local peak pressures exceed the half-maximum peak pressure. Both focal volumes are highly axially (z-direction) elongated in the order of a factor of 10 over their lateral extent (r-radially). The ellipsoidal focal volume may be found by Vel=43πr2z2, however a spheroidal approximation of Vsp=43πr3 is more suitable for in vitro experimentation where samples rarely can take advantage of the lateral extent of the acoustic field. In further restricting the treatment zone to half of the spatial extent of the −6 dB zone for additional consistency in the pressure fields [17] and using a typical lateral focus size of an electromagnetic applicator of r = 2 mm, we obtain a maximum sample volume which can be uniformly treated to be $V_{sample}=34\;\mu L$. For experiments involving either cell suspension or cell pellets, it is unrealistic to contain the entire sample to this volume. In order to homogeneously treat the entire desired sample using this applicator technology, it must be actively moved within the acoustic field. This also introduces a statistical parameter to the otherwise highly reproducible fields. While the stochastic nature is an inherent challenge for electrohydraulic applicators, sample agitation may not be necessary for electrohydraulic applicators as the focal field inherently wanders, resulting in a much larger treatment area. However, beyond these limitations, additional sample agitation may still be necessary to assure homogenous treatment of all cells. At this point, the acoustical fields and the associated treatment parameters for all technologies must be evaluated statistically, as introduced so far. Consequently, minimal pulse numbers to provide a uniform sample treatment must be determined as a function of pulse-to-pulse variability of the applicator and fractional volume treated per pulse.

We have illustrated that ASC lowering/sedimentation is a viable strategy in effectively targeting a majority of cells within a defined treatment zone when cell suspensions are required to conduct the experiment. While this approach allows for spatial confinement of the sample, it is noteworthy that sedimentation proceeds slowly over one hour and re-agitation has to be carefully monitored. To allow for cell culture preparation and feasibility, we propose a minimal sample preparation time of 40 min to let a significant fraction of the ASC to sediment and accumulate at the bottom of the Falcon tube. Once satisfactory sedimentation is achieved, we saw no active re-mixing of the sample during ESWT even at the highest energy settings. If the experimental setup allows it, a mild centrifugation step could also allow for sedimentation of the cells to the focal volume.

The following strategy is devised to give researchers the highest level of flexibility when designing their experiments without forgoing sound physical characterization:

- Use simple degassed water tanks; directly mounted applicator (no additional membranes), no necessity for wave breaker or absorbers.
- Determine the applicator’s main axial direction using hydrophone measurement. Line-of-sight optical alignment between marked points on the tank may be used for sample positioning.
- In situ hydrophone measurements to obtain physical pulse characterization, including any relevant statistical data, are affordable and easily implemented.
- A 40 min sedimentation time or centrifugation of cells in the Falcon tube before treatment. Subsequent ESWT exposure does not result in significant mixing of the sample.
- Treatment parameters should be first evaluated in situ and adjusted for minimal pulse counts and complete volumetric treatment of the sample (limited mixing of the sample is to be expected and agitation may be necessary).

## 4. Materials and Methods

### 4.1. Generation of Shockwaves

The underlying mechanism of all types of shock wave generating devices is the conversion of electrical into acoustic energy. The nature of this conversion can be designed such that the resultant high-pressure wave takes on shockwave characteristics. In the electrohydraulic approach, an electric spark discharge is created across two electrodes. A resultant supersonic expanding plasma bubble compresses the surrounding water resulting in a spherically expanding shock wave which is subsequently refocused outside the applicator. Inherently random spark paths, electrode shape and deterioration, and local cavitation bubbles result in a high shot-to-shot variability with this kind of shock wave generation. In contrast, in electromagnetic devices, a strong electric current is used to displace a flat or cylindrical metallic membrane, which in turn, creates highly consistent and localized pressure waves through acoustic lenses or hyperbolic reflectors.

Electromagnetic, unlike electrohydraulic devices, only build up shockwaves in the vicinity of their significantly smaller focal volumes and otherwise behave as linear pressure waves. Furthermore, generated tensile waves, especially when operated at low energy settings, are much more pronounced. However, they do not exhibit the pronounced shot-to-shot variability associated with the stochastic spark-based shock wave generation of electrohydraulic devices. This allows for the systematic investigation of the energy and pressure-dependent impact of utilized cell containers. Experimental measurement data is presented from an electrohydraulic applicator (OP 155 connected to an Orthogold 100 device, MTS Medical, Konstanz, Germany) and an electromagnetic device (DUOLITH SD1 «ultra», Storz Medical, Tägerwilen, Switzerland) with the “Sepia” handpiece. Each device was attached to a water bath and coupled directly to the water by only the standard membrane.

### 4.2. Water Bath

**Figure 10:**
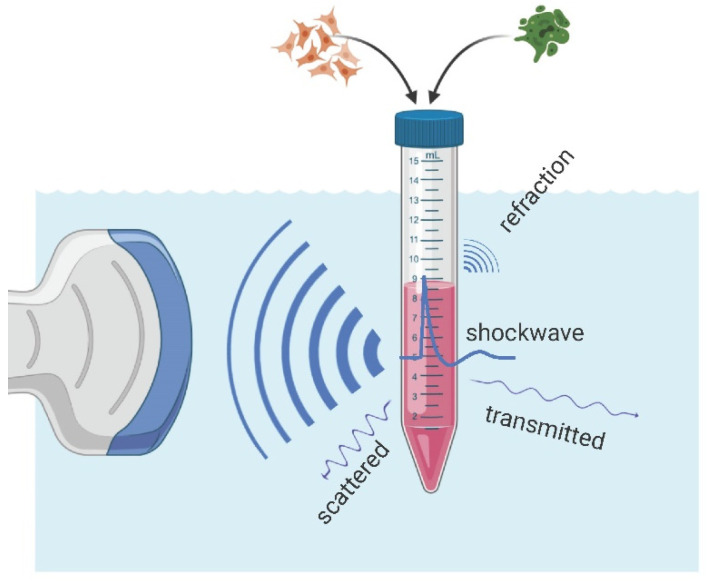
Schematic overview of an in vitro experimental setup. A shockwave applicator (left) generates a focusing acoustic wave which creates a strong shockwave inside the treatment zone of a biological sample. The transmitted wave is attenuated throughout by complex scattering and absorption processes resulting in important refraction phenomena in the vicinity of air–liquid-interfaces.

The key aims of the in vitro experiments are to either establish a correlation between physical shockwave parameters and biological responses of the studied cell and/or tissue samples or to apply a well understood and replicable treatment in the investigation of causation. This requires a well-described environment that should also resemble the physical characteristics of the clinical application. While a homogenous medium, free of reflection or refraction (free field), is desirable, the proximity effects of bones and tissue–air interfaces should be systematically investigated. See Figure 10 for a schematic overview of an in vitro water bath setup.

In the use of strongly and weakly focusing reflector geometries, the strongest pressures are only found in the proximity of the focal volume. Those peak pressures and associated high energy densities are created by the superposition of individual incident wavelets. After they pass through the focal area, they continue to radially diverge, losing intensity at a rate ∝1r2 in the far field. We have chosen a cuboid water bath without refocusing geometries with an axial dimension of 20 cm for an applicator focal length of 4 cm. This assures the original pulse will decay past the initial focus due to absorption within the medium and will have dissipated well before the application of the next pulse.

Reflections of propagating wave-pulses alter the acoustic sound field add further to the complexity of correlating with physical parameters in the area of interest. Shockwaves continually move through a medium only subject to a frequency-dependent absorption, which may alter its waveform or speed of propagation due to the non-linearity of the speed of sound. We use vacuum ejector degassed room temperature water at <10% dissolved oxygen saturation to reduce the likelihood of cavitation and improve pulse transmission. However, any change in impedance of the medium will result in a partial reflection and transmission of the wave front, impacting the pressures and directions of the propagating waves.

Of particular concern are reflections on air interfaces (high to low impedance discontinuity) in the vicinity of the wave’s acoustical path. These lead to an almost complete reflection of the incident wave, which incurs a phase inversion upon reflection. A consequentially enhanced tensile part of the wave, however, would only occur in the immediate vicinity of the interface (pressure zone) as no resonance effects occur with these single pulses, as we would see with continuous waves (i.e., ultrasound). We can estimate the size of this zone based on the pulse duration d which consists of a brief compressive pressure containing the shock front and a longer subsequent tensile pressure and tail oscillations. Using a conservative estimate of d= 5 μs, we estimate the size of the pressure zone where we would expect enhanced tensile forces to extend no larger than 7.5 mm from the interface.

An in vitro setup consisting of a water bath in which the biological samples are submerged has turned out as an ideal candidate to meet all challenges mentioned above. Short focal lengths (<10 cm) only require small tank sizes to achieve an effective free field zone, as any reflections occurring on the bath boundaries will be weak and, in the absence of a refocusing tank-geometry, neglectable. As such, there is no additional need for absorbing boundary layers or “wave-breakers” to achieve a genuinely reflection-free environment. Any samples and containers of comparable acoustic impedance can now be studied in an approximate free field. A similar approach has been adapted for an in vivo mouse model using a “diver box” to create a mostly reflection-free sound field [28].

### 4.3. Sound Measurements

The international standard IEC 61846 allows for the use of either laser optic fibers or polyvinylidene fluoride (PVDF) hydrophones in the characterization of shock wave pressure fields. In opting for PVDF hydrophones, we are able to obtain repeated, localized in situ measurements at high pulse repetition frequencies. Pressure measurements were recorded with a Müller-Platte Needle Probe PVDF hydrophone (Dr. Müller Instruments, Oberursel, Germany) with a pressure range from −10 to 150 MPa and bandwidth from 0.3 to 11 MHz ± 3.0 dB Data collection was done using a digital storage oscilloscope (4-channel, 100 MHz bandwidth, DS1104Z Plus, Rigol, Beijing, China). All hydrophone measurements for the characterization of the in situ sound fields are taken inside a water bath which was mounted onto a compound table to enable centering of the hydrophone in the applicator’s focal zone with regards to all three spatial dimensions.

### 4.4. Imaging

Beyond point-wise hydrophone measurements, there is no direct way of quantifying the entire acoustic sound field. Quasi-quantitative Schlieren-based visualization techniques have emerged but are not yet a viable option. While for electromagnetic and piezo devices, a volumetric mapping may be possible, the stochastic nature of electrohydraulic devices prohibits such an approach. Shadowgraph, Schlieren, and interferometry imaging methods provide a qualitative look at wave propagation and sound field geometries. Resultant images are plane-specific or integrative over the volume and provide no information on local pressures. However, they are a great tool for investigating the effects of reflection and refraction of samples that are introduced into the free-field water bath. In clearly being able to identify regions of concern in vitro, researchers may modify their setup or provide further geometric acoustic parameters.

In our setup, we employed an optical phase contrast detection (OPCD) method, wherein the resultant contrast is proportional to the integrated pressure over the depth of the viewing plane. An optically expanded diode-pumped solid-state laser (wavelength 527 nm, 8 ns pulse duration, Laser-compact group, Moscow, Russia) illuminated the water bath perpendicular to the main axis of the shockwave generator. The pressure variations of the shockwave pulse resulted in a proportional phase shift of the irradiating light. A subsequent projection onto an optical phase plate provided a measurable intensity modulated image of the integrated field which was recorded by a 2D array detector using a 14-bit CCD camera (Type: pco.2000s, PCO AG, Munich, Germany). Further details of the setup can be found in [29,30].

### 4.5. Cell Preparation

In a controlled study of biophysical phenomena, special considerations have to be made to establish a clear correlation of physical and biological parameters. This can become particularly challenging if cell models cannot be reduced in size to approximately half the −6 dB focal volume where a uniform acoustic field approximation may be made [17]. Using cell sedimentation as a means of localizing cells within the focal volume, a suspected subsequent re-mixing during ESWT application was explored by monitoring cell distribution within filled Falcon tubes (Greiner, Kremsmünster, Austria).

Our in vitro setup utilized human adipose-derived stromal cells (ASC) isolated from the stromal vascular fraction (SVF). In addition to ASCs, the SVF included a variety of different cell types from the surrounding tissue; endothelial cells, pericytes, erythrocytes, lymphocytes, monocytes, fibroblasts, macrophages, and adipocytes [31,32]. After liposuction of subcutaneous adipose tissue, the lipoaspirate was cleaned and processed from the Red Cross Blood Transfusion Service of Upper Austria in Linz, to isolate the SVF. The collection of human adipose tissue was approved by the local ethical board with written patient consent. Subcutaneous adipose tissue was obtained during routine outpatient liposuction procedures under local tumescence anesthesia. Cells were procured following the established protocol found in [33]. The SVF is stored at −80 degree until expansion.

In preparation for use in sedimentation experiments 1.0 × 10^6^ cells were thawed in a medium flask (T75, Greiner, Kremsmünster, Austria). Cells were used in passage 4 and were cultivated under standard cell culture conditions (37 °C, 5% CO_2_) in endothelial cell growth medium BulletKit (EGM-2; Lonza, Basel, Switzerland) supplemented with 5% fetal calf serum (FCS). On day three, the cells reached a confluency of over 70%. After harvesting the cells, we resuspended them to a concentration of 5 × 10^5^ cells/5 mL and transferred them into a 15 mL Falcon tube.

## 5. Conclusions

A successful search of a causal bio-physical mechanism requires a reliable and complete spatial and temporal description of physical shockwave parameters. We illustrate the stochastic nature of a low-mid energy electrohydraulic device which necessitates a statistical approach to in vitro setup characterizations. Device-specific free-field measurements are inadequate to capture actual local sound parameters, which are affected by local variations in impedance brought about by samples, containers, and possibly present air interfaces. This can be addressed by directly measuring the applied shockwaves within the sample, thus providing a more complete physical description. PVDF hydrophones in conjunction with suitable storage oscilloscopes provide a simple and affordable means to ascertain the desired physical parameter. Based on an understanding of the overall sound fields, researchers may devise an experimental treatment plan for applied energies, pulse repletion frequencies, sample volumes, and minimal treatment pulse numbers to assure statistical comparability.

Final experimental plans are best evaluated additionally by clinicians to assure clinical relevance on the one hand and physicists for basic science guidance on the other hand. By following our suggestions, experimental data in the field of shockwave therapy will become increasingly reproducible and comparable. As the search for an underlying mechanism and associated physical properties continues, we remain confident that our dedicated research community will greatly benefit from thorough and robust physical characterizations of in vitro experiments.

## Data Availability

The data presented in this study are available on request from the corresponding author. The data are not publicly available due to potential conflicts of confidentiality.
